# Best Prediction of the Additive Genomic Variance in Random-Effects Models

**DOI:** 10.1534/genetics.119.302324

**Published:** 2019-08-05

**Authors:** Nicholas Schreck, Hans-Peter Piepho, Martin Schlather

**Affiliations:** * Research Group on Stochastics and its Applications, School of Business Informatics and Mathematics, University of Mannheim, 68159, Germany; † Biostatistics Unit, Institute of Crop Science, University of Hohenheim, 70593 Stuttgart, Germany; ‡ Animal Breeding and Genetics Group, Center for Integrated Breeding Research, University of Goettingen, 37075, Germany

**Keywords:** best prediction, genetic variance, quantitative genetics, genomic variance, random-effects models, BLUP, whole-genome regression

## Abstract

The additive genomic variance in linear models with random marker effects can be defined as a random variable that is in accordance with classical quantitative genetics theory. Common approaches to estimate the genomic variance in random-effects linear models based on genomic marker data can be regarded as estimating the unconditional (or prior) expectation of this random additive genomic variance, and result in a negligence of the contribution of linkage disequilibrium (LD). We introduce a novel best prediction (BP) approach for the additive genomic variance in both the current and the base population in the framework of genomic prediction using the genomic best linear unbiased prediction (gBLUP) method. The resulting best predictor is the conditional (or posterior) expectation of the additive genomic variance when using the additional information given by the phenotypic data, and is structurally in accordance with the genomic equivalent of the classical additive genetic variance in random-effects models. In particular, the best predictor includes the contribution of (marker) LD to the additive genomic variance and possibly fully eliminates the missing contribution of LD that is caused by the assumptions of statistical frameworks such as the random-effects model. We derive an empirical best predictor (eBP) and compare its performance with common approaches to estimate the additive genomic variance in random-effects models on commonly used genomic datasets.

THE additive genetic variance is defined as the variance of the breeding value (BV) and is the most important determinant of the response of a population to selection (Falconer and Mackay 1996). The additive variance can be estimated from observations made on the population and is a principal component of the (narrow-sense) heritability, which is one of the main quantities of interest in many genetic studies (Falconer and Mackay 1996). The heritability is eminent, among other things, for the prediction of the response to selection in the breeder’s equation (Piepho and Moehring 2007; Hill 2010). Although nonadditive genetic variation exists, most of the genetic variation is additive, such that it is usually sufficient to investigate the additive genetic variance (Hill *et al.* 2008). More specifically, epistasis is only important on the gene level but not for the genetic variance (Hill *et al.* 2008), and Zhu *et al.* (2015) show that, for human complex traits, dominance variation contributes little. Nevertheless, linkage disequilibrium (LD) is an important factor, especially when departing from random mating and Hardy-Weinberg equilibrium, which is often the case in animal breeding (Hill *et al.* 2008; L. Dempfle, personal communication).

The additive genomic variance is defined as the variance of a trait that can be explained by a linear regression on a set of markers (de los Campos *et al.* 2015).

Many authors have been chasing what is sometimes coined “missing heritability” (Maher 2008), which means that only a fraction of the “true” genetic variance can be captured by regression on influential markers. Initially, researchers have used genome-wide association studies (GWAS) in order to find quantitative trait loci (QTL) by single-marker fixed-effect regression combined with variable selection. After having added the estimated corresponding genomic variances of the single statistically significant loci, they asserted that they could only account for a fraction of the “true” genetic variance. For instance, Maher (2008) found that only 5% instead of the widely accepted heritability estimate of 80% of human height could be explained.

Golan *et al.* (2014) state that the “true” genetic variance is generally underestimated when applying variable selection, *e.g.*, GWAS, to genomic datasets which are typically characterized by their high dimensionality, where the number of variables (markers) *p* is much larger than the number of observations *n*. It is well known that a lot of traits are influenced by many genes, and that at least some loci with tiny effects are missed when using variable selection or even single-marker regression models.

Consequently, Bernardo (1994) decided to fit all [restriction fragment length polymorphism (RFLP-)] markers in maize jointly using genomic best linear unbiased prediction (gBLUP), where he assumes the marker effect vector to be random. In animal breeding, Meuwissen *et al.* (2001) used Bayesian approaches (BayesA and BayesB) to fit all markers jointly in order to predict BVs.

Then, Yang *et al.* (2010) estimated the genomic variance in an approach that they termed genome-wide complex trait analysis genomic restricted maximum likelihood (GCTA-GREML) (Yang *et al.* 2011). They showed that quantifying the combined effect of all single-nucleotide polymorphisms (SNPs) explains a larger part of the heritability than only using certain variants quantified by GWAS methods. They illustrate their results on a dataset on human height by pointing out that they could explain a heritability, also termed “chip heritability” (Zhou *et al.* 2013), of ∼45%. They concluded that the main reason for the remaining missing heritability was incomplete LD of causal variants with the genotyped SNPs, which refers to the general difference between the genetic variance and the genomic variance (Powell *et al.* 2010; de los Campos *et al.* 2015). However, the GCTA-GREML approach can be biased upwards as well as downward (Wolc *et al.* 2013; de los Campos *et al.* 2015; Fernando *et al.* 2017a; Lehermeier *et al.* 2017).

Recently, there has been a general discussion whether estimators for the genomic variance account for LD between markers, which is defined as the covariance between the marker genotypes (Bulmer 1971). Some authors argue that estimators similar to GCTA-GREML lack the contribution of LD (Krishna Kumar *et al.* 2016a,b; Lehermeier *et al.* 2017), whereas others (Yang *et al.* 2016) resolutely disagree. More specifically, Krishna Kumar *et al.* (2016a,b) state that, in GCTA-GREML, the contributions of the *p* markers to the phenotypic values are assumed to be independent normally distributed random variables with equal variances. Thus, they claim that the random contribution made by each marker is not correlated with the random contributions made by any other marker, which leads to a negligence of the contribution of LD to the additive genomic variance.

In a study on the model plant *Arabidopsis thaliana* (1001 Genomes Consortium 2016), Lehermeier *et al.* (2017) use Bayesian ridge regression (BRR) to relate the phenotype flowering time to the genomic data. They use an estimator (termed M2) based on the posterior distribution of the marker effects obtained by Markov Chain Monte Carlo (MCMC) methods and show that this estimator explains a larger proportion of the phenotypic variance than the estimator, termed M1, based on gBLUP (VanRaden 2008; Yang *et al.* 2010, 2011). Lehermeier *et al.* (2017) argue that the reason for the better performance of the Bayesian estimator for the additive genomic variance [already mentioned in Sorensen *et al.* (2001), Fernando and Garrick (2013), Zhou *et al.* (2013), Fernando *et al.* (2017b)] is the explicit inclusion of LD.

We show that the additive genomic variance in linear models with random marker effects (RME) can be defined as a random variable. Based on this premise, we propose a novel predictor of the additive genomic variance and place existing estimators in a joint framework permitting comparison with the new predictor. We contribute to the solution of many of the above mentioned controversies by reviewing common approaches to estimate the additive genomic variance, *e.g.*, GCTA-GREML, and show that they estimate the unconditional (or prior) expectation of the random additive genomic variance. Combined with the assumptions on the unconditional distribution of the marker effects in the gBLUP-method, this leads to an insufficient adaptation to the data and a negligence of the contribution of LD.

We introduce a novel best prediction approach for the additive genomic variance in both the current and the base population, *i.e.*, we use the conditional (or posterior) expectation of the random additive genomic variance given the additional information by the phenotypic values for an improved adaptation to the data. We decompose the best predictor into the GCTA-GREML estimator and a function for the contribution of marker LD, which determines whether GCTA-GREML is biased up- or downward. The best predictor is structurally in accordance with the genomic equivalent of the additive genetic variance from classical quantitative genetics, *i.e.*, it explicitly includes the contribution of LD. We propose an empirical best predictor (eBP), and illustrate our theoretical results on several commonly used genomic datasets.

## Materials and Methods

### Linear models

The connection of the *n*-vector *y* of phenotypic values and the *n*-vector *g* of genomic values is given by

$$y=\mu 1_{n}+g+\varepsilon ,$$ (1)

where μ denotes a fixed intercept, $1_{n}:={\left(1,\ldots ,1\right)}^{⊤}$ is a *n*-row-vector containing 1s, $\varepsilon ∼N\left(0,\sigma_{\varepsilon }^{2}I_{n}\right)$ denotes environmental deviations, and $I_{n}$ is the identity matrix of dimension *n*.

The statistical model that is probably most popular in genomic applications is the random-effects model (REM), in which the genomic values are assumed to be normally distributed

$$g∼N\left(0,\sigma_{g}^{2}G\right),$$ (2)

where **G** is a variance-covariance matrix and $\sigma_{g}^{2}>0$ is the variance component of the vector of genomic values *g*.

In the following, we assume that the genome is mapped with p∈ℕ markers and we denote by **X** the n×p design matrix coding the genotypes of the markers.

In this framework, the n×n-matrix **G** introduced in Equation 2 is called the genomic relationship matrix (GRM), and is most commonly defined as

$$G:=PXX^{⊤}P/c,$$ (3)

where $P:=I_{n}-1_{n}1_{n}^{⊤}/n$ is the idempotent n×n-matrix used for column-wise mean-centering, and $c:=2\sum p_{j}\left(1-p_{j}\right)$, where $p_{j}$ denotes the frequency of the minor allele at marker *j* (VanRaden 2008).

Then, the genomic values *g* can be separated into the coded genotypes of the single markers **X** and their corresponding *p*-vector *β* of marker effects such that

$$g\overset{\text{d}}{=}PX\beta ={[\sum_{j=1}^{p}\left(x_{ij}-{\bar{x}}_{\cdot j}\right)\beta_{j}]}_{i=1,\ldots ,n},$$ (4)

holds in distribution, where $\bar{x}._{j}:=\sum_{i=1}^{n}x_{ij}/n=2p_{j}$ for j=1,…,p. The *p* individual components of the marker effect vector *β* are drawn at random from a common fixed normal distribution for each marker genotype:

$$\beta_{j}∼N\left(0,\sigma_{\beta }^{2}\right),j=1,\ldots ,p$$ (5)

with positive variance component $\sigma_{\beta }^{2}:=\sigma_{g}^{2}/c$.

Model (1) is called linear equivalent model (Henderson 1984) to the “standard” additive linear regression model

$$y=\mu 1_{n}+PX\beta +\varepsilon .$$ (6)

The centering matrix **P** in Equations 4 and 6 is mandatory for the equivalence of models (1) and (6), and to be able to consistently apply model (6).

The restriction of the column-means of the marker genotypes in model (6) to be 0 guarantees that the sample mean of the genomic values in Equation 4 equals 0 $\left(\bar{g}:=\frac{1}{n}1_{n}^{⊤}g=0\right)$. This ensures the uniqueness of the definition of the vector *g* of genomic values in Equation 4. Otherwise, a different coding of the marker genotypes leads to different genomic values *g*.

Inferences on quantities based on genomic data in model (1) and (6) are often performed with the gBLUP method (Bernardo 1994). Model (6) allows for marker-specific investigations and inferences on the genomic contribution to the phenotypic values, whereas estimation of parameters in model (1) has computational advantages. For additional information on the gBLUP-method, we refer to Appendix *Genomic Best Linear Unbiased Prediction*.

Model (6) is a realization of *n* draws from the underlying data-generating process of the (mean-centered) marker genotypes $\left(X_{1},\ldots ,X_{p}\right)$ (Bühlmann and van de Geer 2011). This distribution, as well as the corresponding genomic values in Equation 4, relate to the current population of individuals.

When we are interested in the genomic values in the corresponding consistent base population, we should take the relationship (correlation) between the individuals into account (Powell *et al.* 2010; Legarra 2016). In what follows, we assume a given n×n relationship matrix **R**. Instead of the genomic values *g* or PXβ, we investigate the uncorrelated genomic values defined by

$$g^{\text{*}}:=R^{-0.5}g=R^{-0.5}PX\beta =:X^{\text{*}}\beta .$$ (7)

These are realizations of *n* draws from the underlying data-generating process $\left(X_{1}^{\text{*}},\ldots ,X_{p}^{\text{*}}\right)$ of marker genotypes in the base population. The sample mean of the genomic values in the base population, $\frac{1}{n}1_{n}^{⊤}g^{\text{*}}$, is usually different from 0.

### Definitions of the genomic variance

The additive genetic variance (in the base population also called genic variance) with respect to a trait is defined as the theoretical variance of the genetic value based on the respective QTLs (Falconer and Mackay 1996). The genomic variance has been described as the variance of the genomic values that can be explained by a linear regression on a set of markers (de los Campos *et al.* 2015). This does not, however, imply a unique formal definition of the genomic variance.

Here, we give an overview of different approaches to define a genomic variance in the framework of the linear models (1) and (6).

Without further assumptions on the nature of the genome, we can define the sample variance

$$s_{g}^{2}:=\frac{1}{n-1}g^{⊤}g=\frac{1}{n-1}\beta^{⊤}X^{⊤}PX\beta =\beta^{⊤}{\hat{\Sigma }}_{X}\beta$$ (8)

of the mean-centered *n*-vector of genomic values g=PXβ (see Equation 4) in the current population (Ould Estaghvirou *et al.* 2013). Here, ${\hat{\Sigma }}_{X}:=X^{⊤}PX/\left(n-1\right)$ defines the sample variance-covariance matrix of the marker genotypes in the current population.

In the base population, we define the sample variance

$$s_{g^{*}}^{2}:=\frac{1}{n-1}{\left(g^{\text{*}}\right)}^{⊤}Pg^{\text{*}}=\frac{1}{n-1}\beta^{⊤}{\left(X^{\text{*}}\right)}^{⊤}PX^{\text{*}}\beta =\beta^{⊤}{\hat{\Sigma }}_{X*}\beta$$ (9)

of the uncorrelated genomic values $g^{\text{*}}=X^{\text{*}}\beta$ (see Equation 7). Here, ${\hat{\Sigma }}_{X*}:={\left(X^{\text{*}}\right)}^{⊤}PX^{\text{*}}/\left(n-1\right)$ defines the sample variance-covariance matrix of the marker genotypes in the base population.

Alternatively, we can define the theoretical variance of the genomic values directly in the REM. The linear model is generated by drawing from the data-generating process of the marker genotypes (representative individual), and the model assumptions in the REM dictate that marker effects are random variables. This gives rise to three different scenarios for the variance of the genomic values in the REM (marker genotypes random, marker effects random, or both random).

The additive genomic variance of a randomly sampled (representative) individual (Gianola *et al.* 2009; de los Campos *et al.* 2015; Fernando *et al.* 2017b) equals

$$\text{Var}\left(X\beta |\beta \right)=\beta^{⊤}{\text{Σ}}_{X}\beta ,$$ (10)

where ${\text{Σ}}_{X}$ denotes the variance-covariance matrix of the marker genotypes.

The variance of a randomly sampled (representative) individual with RME is given by

$$\text{Var}\left(X\beta \right)=\sigma_{\beta }^{2}\text{tr}\left({\text{Σ}}_{X}\right).$$ (11)

This is not the additive genomic variance (Gianola *et al.* 2009; de los Campos *et al.* 2015).

The variance of a randomly sampled trait averaged over individuals with fixed genotypes **X** equals

$$\frac{1}{n}\text{tr}\left(\text{Cov}\left(X\beta |X\right)\right)=\sigma_{\beta }^{2}\frac{n-1}{n}\text{tr}\left({\hat{\Sigma }}_{X}\right)\approx \sigma_{\beta }^{2}\text{tr}\left({\hat{\Sigma }}_{X}\right),$$ (12)

and does not equal the additive genomic variance.

We derive the equalities in Equations 10–12 in more detail in the Appendix *Theoretical Variances of the Genomic Values in the REM*. These quantities refer to the genotypes in the current population. We can apply the same definitions in the base population by considering the data-generating process of the genotypes in the base population (exchange *X* by $X^{\text{*}}$).

In Table 1, we give an overview of the different possibilities to define the variance of the genomic values in the REM.

**Table 1:** Overview of different definitions of the variance of the genomic values in the current population and their expression in the random-effects model

Variance of genomic values			
Sample variance	Theoretical variance		
$s_{g}^{2}$	Var(Xβ)	tr(Cov(Xβ|X))	Var(Xβ|β)
$\beta^{⊤}{\hat{\Sigma }}_{X}\beta$	$\sigma_{\beta }^{2}\text{tr}\left({\text{Σ}}_{X}\right)$	$n\sigma_{\beta }^{2}\text{tr}\left({\hat{\Sigma }}_{X}\right)$	$\beta^{⊤}{\text{Σ}}_{X}\beta$

Analogous quantities for the base population can be obtained by exchanging *X* by $X^{\text{*}}$. The sample variance $s_{g}^{2}$ and the theoretical variance Var(Xβ|β) define the sample and theoretical version of the additive genomic variance.

In actual applications, we have to replace ${\text{Σ}}_{X}$ in Equation 10 by its estimator ${\hat{\Sigma }}_{X}$. Consequently, the sample variance (Equation 8) as well as the theoretical variance (Equation 10) effectively represent the additive genomic variance, *i.e.*, the genomic equivalent of the additive genetic variance (Bulmer 1971; Falconer and Mackay 1996), in the current population. In the following, we do not explicitly distinguish between the sample or the theoretical version of the variance, and will speak only of the additive genomic variance.

From now on, we focus on the estimation of the additive genomic variance in the general form

$$s_{g,B}^{2}:=\frac{1}{n-1}g^{⊤}Bg=\frac{1}{n-1}\beta^{⊤}X^{⊤}PBPX\beta ,$$ (13)

which is a non-negative quadratic form of the genomic values. By specifying the positive semidefinite and symmetric n×n-matrix **B**, we determine whether the genomic variance refers to the current population $\left(B=I_{n}\right)$ (see Equation 8), or the base population $\left(B=R^{-0.5}PR^{-0.5}\right)$ (see Equation 9). Because the randomness of the marker genotypes is not explicitly necessary to derive Equation 13, we can easily express all results in terms of the genomic values *g* defined in the equivalent model.

In the framework of the REM, the marker effects *β* in model (6) and the genomic values *g* in model (1) are random variables. Consequently, the additive genomic variance in Equation 13 is also a random variable, and, as such, has to be predicted in an optimal way before finally being estimated [in the same way that the RME *β* are predicted by the BLUP and then estimated by the eBLUP (Kackar and Harville 1984; Jiang 1999)].

First, we will show that estimators for the unconditional expectation of Equation 13, like GCTA-GREML, are of the form of Equations 11 and 12, and, therefore, do not estimate the additive genomic variance.

Then, we introduce the (frequentist) best predictor for the additive genomic variance $s_{g,B}^{2},$ and show that this approach maintains the structure of the additive genomic variance (Equation 13)—the genomic equivalent of the additive genetic variance.

### The expectation of the additive genomic variance

The expectation of the random variable $s_{g,B}^{2}$ in Equation 13 minimizes the quadratic form

$$E\left[{\left(s_{g,B}^{2}-a\right)}^{2}\right],$$

with respect to all real numbers *a*, *i.e.*, $\tilde{a}:=E\left[s_{g,B}^{2}\right]$ is the best approximation of $s_{g,B}^{2}$ in the absence of additional information (van der Vaart 2007). The unconditional (or prior) expectation of $s_{g,B}^{2}$ equals

$$\begin{array}{l} E\left[s_{g,B}^{2}\right]=E\left[\frac{1}{n-1}\beta^{⊤}X^{⊤}PBPX\beta \right]\\ =\frac{1}{n-1}\text{tr}\left(X^{⊤}PBPXE\left[\beta \beta^{⊤}\right]\right)\\ \overset{\left(5\right)}{=}\frac{1}{n-1}\sigma_{\beta }^{2}\text{tr}\left(X^{⊤}PBPX\right)\\ \overset{\left(3\right)}{=}\frac{1}{n-1}\sigma_{g}^{2}\text{tr}\left(BG\right) \end{array}$$ (14)

because of the properties of the trace.

For the additive genomic variance in the current population, $s_{g}^{2}$, we choose $B=I_{n}$ in Equation 14 and obtain

$$V:=E\left[s_{g}^{2}\right]=\sigma_{\beta }^{2}\text{tr}\left({\hat{\Sigma }}_{X}\right)$$ (15)

in model (6) or

$$V=\frac{1}{n-1}\sigma_{g}^{2}\text{tr}\left(G\right)$$ (16)

in the equivalent model (1). Unconditional expectations of the form *V* for the additive genomic variance are considered in, for example, Ould Estaghvirou *et al.* (2013).

For the additive genomic variance in the base population, $s_{g^{*}}^{2}$, we choose $B=R^{-0.5}PR^{-0.5}$ in Equation 14 and obtain

$$V^{\text{*}}:=E\left[s_{g*}^{2}\right]=\sigma_{\beta }^{2}\text{tr}\left({\hat{\Sigma }}_{X^{\text{*}}}\right)$$ (17)

in model (6) or

$$V^{\text{*}}=\frac{1}{n-1}\sigma_{g}^{2}\text{tr}\left(PR^{-0.5}GR^{-0.5}\right)$$ (18)

in the equivalent model.

Often (VanRaden 2008; Yang *et al.* 2010, 2011; Speed *et al.* 2012; Vinkhuyzen *et al.* 2013; Legarra 2016), the matrix **R** used for the transformation to the base population is assumed to be the GRM **G** defined in Equation 3. Then, the unconditional expectation $V^{\text{*}}$ of the additive genomic variance simplifies to

$$V_{s}^{*}:=\frac{1}{n-1}\sigma_{g}^{2}\text{tr}\left(P\right)=\sigma_{g}^{2},$$ (19)

and the variance component $\sigma_{g}^{2}$ from the gBLUP-method is considered as the additive genomic variance in the base population. We have shown, however, that $\sigma_{g}^{2}$ is only the unconditional expectation of the additive genomic variance.

We recommend caution when using the simplification in Equation 19 because the GRM **G** is in general singular (because **P** is singular), and therefore $G^{-1}$ is not well defined.

We emphasize that only the diagonal elements of the sample variance-covariance matrix (${\hat{\Sigma }}_{X}$ or ${\hat{\Sigma }}_{X^{\text{*}}}$) of the marker genotypes influence the unconditional expectations *V*, $V^{\text{*}},$ and $V_{s}^{*}$ of the additive genomic variance. The model assumptions in the REM dictate the matrix $E\left[\beta \beta^{⊤}\right]$ to be diagonal, which leads to a negligence of the off-diagonal elements of ${\hat{\Sigma }}_{X}$ or ${\hat{\Sigma }}_{X^{\text{*}}}$ in Equation 14. The covariances (LD) between the marker genotypes are not included, and *V*, $V^{\text{*}},$ and $V_{\text{s}}^{*}$ are of the same form as Var(Xβ) in Equation 11 and $\frac{1}{n}\text{tr}\left(\text{Cov}\left(X\beta |X\right)\right)$ in Equation 12. This implies that the unconditional expectation of the random additive genomic variance $s_{g,B}^{2}$ is structurally not fully in accordance with the (random) additive genomic variance in Equation 13.

Explicit formulae for the estimation of the unconditional expectations *V*, $V^{\text{*}},$ and $V_{\text{s}}^{\text{*}}$ will be given in the Appendix *Estimation of the Additive Genomic Variance in the REM*.

### Best prediction of the additive genomic variance

The unconditional (prior) expectation of $s_{g,B}^{2}$ in Equation 14 is strongly influenced by the model assumption on the marginal distribution of the marker effects, and does not use additional information given by the phenotypic values *y* in model Equations 1 and 6. Estimation procedures for the unconditional expectation [which include *e.g.*, restricted maximum likelihood (REML)], however, make use of the data *y* to a certain extent.

By contrast, the conditional (posterior) expectation, given the phenotypic values *y*, makes best use of the information provided by *y* even before the final estimation is performed.

Generally, the conditional (or posterior) expectation of a random variable *Z* (in our case $Z=s_{g,B}^{2}$) given the knowledge of the random vector *Y* is defined as the “best prediction” (Searle *et al.* 1992; van der Vaart 2007) of the random variable *Z*. The best predictor

BP(Z):=E[Z|Y] (20)

is the unique function $g_{0}\left(Y\right)$ that minimizes the mean square error of prediction

$$E\left[{\left(Z-g\left(Y\right)\right)}^{2}\right]$$

over all functions in *Y*, *i.e.*, the conditional expectation is the projection (closest element in a given set of functions) of *Z* onto the linear space of all functions in *Y* (Searle *et al.* 1992; van der Vaart 2007).

The best predictor in Equation 20 is, by definition, an unbiased predictor for the random variable *Z* and $g_{0}\left(Y\right)$ maximizes the correlation Cor(Z,g(Y)), *i.e.*, we can replace the target random variable *Z* by the best predictor defined in Equation 20 in an optimal way (Searle *et al.* 1992). Instead of inferring the unobservable target random variable, we conduct inferences on the best predictor. Because the best predictor has realized in a given dataset (Y=y), it is estimable (Searle *et al.* 1992).

In the following, we introduce a novel approach of considering the frequentist best predictor instead of the unconditional expectation for the random additive genomic variance $s_{g,B}^{2}$ in Equation 13. We proceed according to Equation 20, and define

$$\text{BP}\left(s_{g,B}^{2}\right):=E\left[s_{g,B}^{2}|y\right]=E\left[\frac{1}{n-1}\beta^{⊤}X^{⊤}PBPX\beta |y\right]$$

$$=\frac{1}{n-1}\text{tr}\left(X^{⊤}PBPXE\left[\beta \beta^{⊤}|y\right]\right)$$

for the given phenotypic values *y*, because **X** is constant, and, therefore, independent of *y*. The matrix of conditional second moments of the marker effects *β* is usually nondiagonal (contrary to $E\left[\beta \beta^{⊤}\right]$), and can be expressed as

$$E\left[\beta \beta^{⊤}|y\right]=\mu_{\beta |y}\mu_{\beta |y}^{⊤}+{\text{Σ}}_{\beta |y}$$

using the BLUP $\mu_{\beta |y}:=E\left[\beta |y\right]$ of the random vector *β*, and the variance-covariance matrix $\sum_{\beta |y}:=\text{Cov}\left(\beta |y\right)$ of *β* given the data *y*. Then, the best predictor equals

$$\begin{array}{l} \text{BP}\left(s_{g,B}^{2}\right)=\frac{1}{n-1}\text{tr}\left(X^{⊤}PBPX\left[\mu_{\beta |y}\mu_{\beta |y}^{⊤}+{\text{Σ}}_{\beta |y}\right]\right)\\ =\frac{1}{n-1}\text{tr}\left(B\left[\mu_{g|y}\mu_{g|y}^{⊤}+{\text{Σ}}_{g|y}\right]\right) \end{array}$$ (21)

where the last equality holds because of the connection

$$\mu_{\beta |y}:=E\left[g|y\right]=E\left[PX\beta |y\right]=PX\mu_{\beta |y}$$

of the BLUPs and the conditional variance-covariance matrices

$$\Sigma_{g|y}:=\text{Cov}\left(g|y\right)=\text{Cov}\left(PX\beta |y\right)=PX{\text{Σ}}_{\beta |y}X^{⊤}P.$$

in models (1) and (6), see also Appendix *Genomic Best Linear Unbiased Prediction*.

For the best predictor of the additive genomic variance in the current population we set $B=I_{n}$ in Equation 21, and obtain

$$W:=\text{BP}\left(s_{g}^{2}\right)=\text{tr}\left({\hat{\Sigma }}_{X}\left[\mu_{\beta |y}\mu_{\beta |y}^{⊤}+{\text{Σ}}_{\beta |y}\right]\right)$$ (22)

in model (6) or

$$W=\frac{1}{n-1}\text{tr}\left(\mu_{g|y}\mu_{g|y}^{⊤}+{\text{Σ}}_{g|y}\right)$$ (23)

in the terminology of the equivalent model (1).

For the best predictor of the additive genomic variance in the base population we set $B=R^{-0.5}PR^{-0.5}$ in Equation 21, and obtain

$$W^{\text{*}}:=\text{BP}\left(s_{g*}^{2}\right)=\text{tr}\left({\hat{\Sigma }}_{X^{\text{*}}}\left[\mu_{\beta |y}\mu_{\beta |y}^{⊤}+{\text{Σ}}_{\beta |y}\right]\right)$$ (24)

in model (6) or

$$W^{\text{*}}=\frac{1}{n-1}\text{tr}\left(PR^{-0.5}\left[\mu_{g|y}\mu_{g|y}^{⊤}+{\text{Σ}}_{g|y}\right]R^{-0.5}\right)$$ (25)

in the terminology of the equivalent model (1).

We note that Equations 24 and 25 have structural similarities with the iterative maximum-likelihood equation for the variance component $\sigma_{\beta }^{2}$ when using the expectation-maximization algorithm (Sorensen and Gianola 2002).

We emphasize that the best predictor of the additive genomic variance in the current population (*W*), as well as in the base population $\left(W^{\text{*}}\right),$ includes the contribution of all elements of the sample variance-covariance matrix of marker genotypes (${\hat{\Sigma }}_{X}$ or ${\hat{\Sigma }}_{X^{\text{*}}}$), and, hence, comprises LD information, contrary to the unconditional expectations *V*, $V^{\text{*}},$ and $V_{\text{s}}^{*}$ of the additive genomic variance from the previous section.

Explicit formulae for the eBPs of the additive genomic variance, as well as a formula for $W_{\text{s}}^{\text{*}}$ (approximate approach using the GRM **G** for transformation to the base population), will be given in the Appendix *Estimation of the Additive Genomic Variance in the REM*. We compare the use of the unconditional expectation and the best predictor for the prediction of the random additive genomic variance in the REM in Table A.1 and Table A.2 in the Appendix.

### Statistical analysis (genomic data)

For an illustration of the theoretical results of the previous sections, we used the mice dataset that comes with the *R*-package BGLR (Pérez and de los Campos 2014). The data originally stem from an experiment by Valdar *et al.* (2006a,b) in a mouse population. The dataset contains the matrix **X** with values in {0,1,2} of p=10,346 polymorphic marker genotypes that were measured in n=1814 mice. The trait (*n*-vector *y*) under consideration was body length (BL). The relationship of the mice is recorded in the n×n pedigree matrix **R**, and is used for the transformation to the base population.

Additionally, we used the publicly available historical wheat dataset that also comes with the *R*-package “BGLR” (Pérez and de los Campos 2014). The data originally stems from CIMMYT’s Global Wheat Program and consists of n=599 lines of wheat, where the trait under consideration was average grain yield. The phenotypes are divided up into four basic target sets of environments designated as Wheat I, Wheat II, Wheat III, and Wheat IV, of which we considered the only first. The dataset contains the matrix of marker genotypes for p=1279 markers as well as a relationship matrix.

Moreover, we analyzed a population of n=1057 fully sequenced *Arabidopsis* lines for which phenotypes and genotypes are publicly available by the effort of the *Arabidopsis* 1001 Genomes project (1001 Genomes Consortium 2016). The lines represent natural inbred lines and we examined the same trait, namely flowering time at 10° (FT10), and the same p=193,697 SNP-markers that were used in Lehermeier *et al.* (2017). For these data, no relationship matrix was available.

We conducted all calculations with the free software R (R Development Core Team 2017). For each dataset, we used the gBLUP-method in the equivalent version (computational advantages) implemented in the *R*-package “sommer” (Covarrubias-Pazaran 2017) to fit a REM. We used REML to obtain estimates (${\hat{\sigma }}_{g}^{2}$ and ${\hat{\sigma }}_{\varepsilon }^{2}$) for the variance components. We also obtained estimates of the best predictor of the genomic effects $\mu_{g|y}$ and the their variance-covariance matrix ${\text{Σ}}_{{\hat{\mu }}_{g|y}}$.

We used this outcome for the estimation of the unconditional expectation *V* and the BP *W* of the additive genomic variance in the current population, as well as the unconditional expectation $V^{\text{*}}$ and the BP $W^{\text{*}}$ for the additive genomic variance in the base population (except for the *Arabidopsis* dataset, where no relationship matrix was available). Although the GRM is not invertible, we will show in the Appendix *Estimation of the Additive Genomic Variance in the REM* how to use the GRM for a transformation to the base population, and to calculate the corresponding unconditional expectation $V_{\text{s}}^{*}$ and the BP $W_{\text{s}}^{\text{*}}$ for the additive genomic variance in the base population.

Detailed information about the calculations and the programming code is provided in the Supplemental Material, File S1.

### Data availability

The authors affirm that all data necessary for confirming the conclusions of this article are represented fully within the manuscript and the supplemental material that has been uploaded to figshare. File S1 contains a detailed description of the estimation of the genomic variances for the gBLUP-method as well as the corresponding *R*-code and its output. Supplemental material available at Figshare: https://doi.org/10.25386/genetics.8114117.

## Results

In the first section of Table 2, we present the estimation results for the unconditional expectation *V* and the best predictor *W* for the additive genomic variance in the current population. In the mice and wheat datasets, $\hat{V}$ exceeds $\hat{W}$, whereas for the *Arabidopsis* data, the eBP is about double the size of the unconditional expectation.

**Table 2:** Estimation results for the unconditional expectation *V* and the best predictor *W* for the additive genomic variance in the current population for the mice, wheat, and *Arabidopsis* datasets

Genomic variance/heritability	Population	Mice	Wheat	*Arabidopsis*
$\hat{V}\left(={\hat{h}}_{V}^{2}\right)$*^a^*	Current	0.3737749	0.6039708	0.47333803
$\hat{V}+{\hat{\sigma }}_{\varepsilon }^{2}$*^b^*		1.0754963	1.1449704	0.54832029
${\tilde{h}}_{V}^{2}:=\hat{V}/\left(\hat{V}+{\hat{\sigma }}_{\varepsilon }^{2}\right)$*^c^*		0.3475371	0.5274990	0.86325098
$\hat{W}\left(={\hat{h}}_{W}^{2}\right)$*^a^*		0.2982787	0.4590001	0.92501779
$\hat{W}+{\hat{\sigma }}_{\varepsilon }^{2}$*^b^*		1.0000002	0.9999998	1.00000005
${\tilde{h}}_{W}^{2}:=\hat{W}/\left(\hat{W}+{\hat{\sigma }}_{\varepsilon }^{2}\right)$*^c^*		0.2982787	0.4590002	0.92501774
${\hat{V}}^{\text{*}}$	Base	0.3704021	3.0621134	−
${\hat{W}}^{\text{*}}$		0.3089758	2.0095836	−
${\hat{V}}_{\text{s}}^{\text{*}}$		0.3639248	1.3158006	0.80762011
${\hat{W}}_{\text{s}}^{\text{*}}$		0.3577692	1.2300300	1.30240520

We also present the corresponding heritabilities with respect to the sample variance of the phenotypic values and with respect to the sum of the additive genomic and residual variance $\sigma_{\varepsilon }^{2}.$ In addition, we depict the estimation results for the unconditional expectation $V^{\text{*}}$ ($V_{s}^{*}$ when using the GRM for the transformation) and the best predictor $W^{\text{*}}$ ($W_{s}^{\text{*}}$ when using the GRM for the transformation) for the additive genomic variance in the base population.

a Heritability with respect to phenotypic sample variance ${\hat{\sigma }}_{y}^{2},$ which has been scaled to 1.

b Alternative definition of the phenotypic variance that depends on the estimate of the genomic variance.

c Alternative definition of the heritability that depends on the alternative definition of the phenotypic variance.

The sample variance of the phenotypic values has been scaled to 1. The sum of $\hat{V}$ and the residual variance is larger than the phenotypic variance for the mice and wheat data but smaller for the *Arabidopsis* data. Technically, it is possible to define the heritability in two ways, namely with respect to the phenotypic variance, and with respect to the sum of the additive genomic variance and the residual variance. The sum of the eBP $\hat{W}$ and the residual variance, however, equals the scaled phenotypic variance of 1 remarkably exactly for all datasets considered.

In the second section of Table 2, we first present the estimation results for the unconditional expectation $V^{\text{*}}$ and the best predictor $W^{\text{*}}$ for the additive genomic variance in the base population using the given relationship matrices for the transformation. For the mice data, ${\hat{V}}^{\text{*}}$ and ${\hat{W}}^{\text{*}}$ are similar to their analogs $\hat{V}$ and $\hat{W}$ in the current population. For the wheat data, however, the estimated unconditional expectation and eBP in the base population are around five times larger than those in the current population, and exceed the sample phenotypic variance in the current population. By this approach, it is not possible to define a heritability in the base population because both the estimate of the residual variance and the phenotypic sample variance refer to the current population.

The estimation results for the unconditional expectation $V_{\text{s}}^{*}$ and the best predictor $W_{s}^{*}$ for the additive genomic variance in the base population using the GRM for the transformation differ from those using the given relationship matrices by a considerable amount. In the mice and wheat data, $V_{\text{s}}^{*}$ is larger than $W_{\text{s}}^{\text{*}}$, whereas, for the *Arabidopsis* data the eBP exceeds the estimated unconditional expectation. This conforms to the behavior of *V* and *W* in the current population.

## Discussion

We have shown that commonly used estimators for the additive genomic variance in the REM with genomic marker data are based on the unconditional expectation of the random additive genomic variance. We have introduced a novel best prediction approach for the random additive genomic variance in both the current and the base population. In the following, we discuss several important implications.

### Current and base population

Common ways of estimating the additive genomic variance focus on the base population. These approaches are independent of the actual current population, and, consequently, valid even if the generations change.

If one aims at the response of a population to selection, however, it might be more meaningful to estimate the additive genomic variance in the actual given population. This implies that the estimation of the genomic variance has to be conducted again when the individuals change. A formal definition of the heritability is best possible in the current population, where the phenotypic and residual variance are estimable.

We have preferred to use given relationship matrices for the transformation of the genomic values to the base population. In the case that such a matrix is not available, we have shown how to use genomic relationship matrices for the transformation, although a formal inversion of GRMs is, in general, not possible.

In Table 2, we illustrate that we can decompose the sample phenotypic variance into the sum of the eBP of the additive genomic variance in the current population and into the estimated residual variance. This is due to the orthogonal projection property of the conditional expectation, which gives the best approximation of the random additive genomic variance. This enables a unique definition of the heritability in the current population. It is never possible, however, to transfer the residual variance to the base population. Consequently, a definition of the heritability in the base population is not straightforward.

### The gBLUP-method and the Bayesian approach

The frequentist gBLUP-method can also be set up in the context of Bayesian regression models (with prior distribution for the effect vector as defined in Equation 5, and uninformative priors for the variance components). Lehermeier *et al.* (2017) considered the additive genomic variance $s_{g}^{2}$ in the current population (see Equation 8), and used BRR to estimate

$$M_{2}:=\frac{1}{M}\sum_{m=1}^{M}{\left({\hat{\beta }}^{\left(m\right)}\right)}^{⊤}{\hat{\Sigma }}_{X}{\hat{\beta }}^{\left(m\right)},$$

where ${\left({\hat{\beta }}^{\left(m\right)}\right)}_{m=1,\ldots ,M}$ denotes MCMC samples from the posterior distribution of *β*. In that approach, Lehermeier *et al.* (2017) estimated the posterior mean of the additive genomic variance $s_{g}^{2}$ in the current population. This approach is the Bayesian equivalent of the (frequentist) empirical version of the best predictor of $s_{g}^{2}$ in Equation 13 in the current population. $M_{2}$ does not describe the genomic variance in the base population, and should not directly be compared with approaches introduced *e.g.*, in Yang *et al.* (2010, 2011). Analogously to the best predictor $W^{\text{*}}$ (see Equation 24), for the genomic variance in the base population, one can consider

$$M_{2}^{\text{*}}:=\frac{1}{M}\sum_{m=1}^{M}{\left({\hat{\beta }}^{\left(m\right)}\right)}^{⊤}{\hat{\Sigma }}_{X^{\text{*}}}{\hat{\beta }}^{\left(m\right)}$$

as the posterior mean of the genomic variance in the base population in Bayesian regression models.

The frequentist gBLUP-method provides a more formal approach to the prediction of the random additive genomic variance in linear models with random effects than the Bayesian approach. It enables the derivation of explicit formulas for the predictors (unconditional expectation and best predictor) of the random additive genomic variance using the standard output of the gBLUP-method, which goes hand-in-hand with a fast implementation of the empirical version of the predictors. The connection between the BLUP $\mu_{\beta |y}$ and its covariance for the RME with the additive genomic variance are clearly visible. This enables us, for instance, to derive the decomposition of the best predictor of the random additive genomic variance into the unconditional expectation and a function for the marker LD in the following section.

### Influence of LD

In the section *Definitions of the genomic variance*, we saw that the (random) additive genomic variance equals

$$s_{g}^{2}=\beta^{⊤}{\hat{\Sigma }}_{X}\beta$$

$$=\sum_{j=1}^{p}\beta_{j}^{2}{\left({\hat{\Sigma }}_{X}\right)}_{jj}+\sum_{i=1}^{p}\sum_{\begin{array}{l} j=1\\ j\neq i \end{array}}^{p}\beta_{i}\beta_{j}{\left({\hat{\Sigma }}_{X}\right)}_{ij}$$

in the current population, and

$$s_{g*}^{2}=\beta^{⊤}{\hat{\Sigma }}_{X^{\text{*}}}\beta$$

$$=\sum_{j=1}^{p}\beta_{j}^{2}{\left({\hat{\Sigma }}_{X^{\text{*}}}\right)}_{jj}+\sum_{i=1}^{p}\sum_{\begin{array}{l} j=1\\ j\neq i \end{array}}^{p}\beta_{i}\beta_{j}{\left({\hat{\Sigma }}_{X^{\text{*}}}\right)}_{ij}$$

in the base population. We emphasize that the variance-covariance matrix of the marker genotypes (marker LD) plays a decisive part in the determination of the additive genomic variances in both the current and the base population. The variances $s_{g}^{2}$ and $s_{g*}^{2}$ are structurally in accordance with the classical additive genetic variance (Bulmer 1971; Falconer and Mackay 1996), which is caused by the genotypes, whereas the genotypic effects are fixed.

In the REM, however, the marker effects are random, with unconditional expectation 0 and unconditional diagonal variance-covariance matrix with equal variances $\sigma_{\beta }^{2}$. As a consequence, the unconditional expectation of the additive genomic variance

$$E\left[s_{g}^{2}\right]=\sigma_{\beta }^{2}\text{tr}\left({\hat{\Sigma }}_{X}\right)$$

in the current population and

$$E\left[s_{g*}^{2}\right]=\sigma_{\beta }^{2}\text{tr}\left({\hat{\Sigma }}_{X^{\text{*}}}\right)$$

in the base population contain only the variances of the marker genotypes in the corresponding population. In addition, the unconditional expectation resembles both the variance of a randomly sampled trait for a randomly sampled individual and the variance of a randomly sampled trait for individual with fixed genotypes, see Table 1 for an overview.

We show in the Appendix *Estimation of the Additive Genomic Variance in the REM* that we can partition the best predictor of the random additive genomic variance $s_{g,B}^{2}$ in the following way:

$$\text{BP}\left(s_{g,B}^{2}\right)=E\left[s_{g,B}^{2}\right]+L\left(y\right),$$

where

$$L\left(y\right)=\sum_{j=1}^{p}{\left[\mu_{\beta |y}\mu_{\beta |y}^{⊤}-{\text{Σ}}_{\mu_{\beta |y}}\right]}_{jj}{\left({\hat{\Sigma }}_{X}\right)}_{jj}$$

$$+\sum_{i=1}^{p}\sum_{\begin{array}{l} j=1\\ j\neq i \end{array}}^{p}{\left[\mu_{\beta |y}\mu_{\beta |y}^{⊤}-{\text{Σ}}_{\mu_{\beta |y}}\right]}_{ij}{\left({\hat{\Sigma }}_{X}\right)}_{ij}$$

in the current population, and

$$L\left(y\right)=\sum_{j=1}^{p}{\left[\mu_{\beta |y}\mu_{\beta |y}^{⊤}-{\text{Σ}}_{\mu_{\beta |y}}\right]}_{jj}{\left({\hat{\Sigma }}_{X^{\text{*}}}\right)}_{jj}$$

$$+\sum_{i=1}^{p}\sum_{\begin{array}{l} j=1\\ j\neq i \end{array}}^{p}{\left[\mu_{\beta |y}\mu_{\beta |y}^{⊤}-{\text{Σ}}_{\mu_{\beta |y}}\right]}_{ij}{\left({\hat{\Sigma }}_{X^{\text{*}}}\right)}_{ij}$$

in the base population $\left({\text{Σ}}_{\mu_{\beta |y}}=\text{Cov}\left(\mu_{\beta |y}\right)\right)$.

The best predictor, therefore, consists of the unconditional expectation of the additive genomic variance (no contribution of LD) and a function that explicitly contains the weighted contribution of marker LD. This function determines whether estimators like GCTA-GREML (unconditional expectation of the random genomic variance in the base population) are biased upwards or downward, *i.e.*, it determines the direction and the magnitude of the bias of GCTA-GREML (this method is based on the assumption that the function *L* constantly equals 0). In addition, we notice that this bias depends not only on the sign of the covariance between the marker genotypes, but both on the sign and the magnitude of the weighted covariances.

When best (conditional) estimators are considered, the inclusion of LD is intrinsic; in that case, discussions about whether or not LD should be included or even optimally included become immaterial.

We emphasize that, contrary to the unconditional expectation, the best predictor maintains the structure of the additive genomic variance $s_{g}^{2}$ and $s_{g*}^{2}$, because the function *L* can be decomposed into the weighted sample variances and covariances of the marker genotypes. Instead of the marker effects, the components of the matrix $\mu_{\beta |y}\mu_{\beta |y}^{⊤}-{\text{Σ}}_{\mu_{\beta |y}}$, which is typically nonzero and nondiagonal, take the part of the weighting factors of the elements of ${\hat{\Sigma }}_{X}$ and ${\hat{\Sigma }}_{X^{\text{*}}}.$ The best predictor maintains the structure of the additive genomic variance in both the current $\left(s_{g}^{2}\right)$ and the base population $\left(s_{g*}^{2}\right),$ and, thus, conforms to the classical genetic variance (Bulmer 1971; Falconer and Mackay 1996).

The difference between the estimators *V* and *W* ($V^{\text{*}}$ and $W^{\text{*}},$$V_{s}^{*}$ and $W_{\text{s}}^{\text{*}}$) is given by the estimated L(y), and can be obtained from Table 2. We notice that the weighted contribution of marker LD is large and positive in the case of the *Arabidopsis* data, whereas in the mice and wheat data, the weighted contribution of marker LD is slightly negative.

To sum up, the application of the unconditional expectation of the additive genomic variance combined with the model assumptions on the marker effects in random effect models cause, at least partially, the missing contribution of LD to the estimated additive genomic variance. This goes hand-in-hand with the critique expressed in Krishna Kumar *et al.* (2016a,b). It is, however, less important when estimating the additive genomic variance in the base population, where the individuals are uncorrelated and less LD persists (although the marker genotypes need not be uncorrelated).

The best prediction approach eliminates the problem of the missing contribution of LD to the additive genomic variance that is caused by mathematical modeling (*e.g.*, the assumptions in the random-effects model).

### Concluding remarks

The variability in the marker genotypes causes the variability in the genomic values in a population of individuals. Hence, the additive genomic variance is induced by the variance of the marker genotypes. In the application of the random-effects model, the marker effects are assumed to be random, which introduces another source of variability, namely a statistical one, in the genomic values.

We have shown that commonly used estimators use the unconditional expectation to handle this statistical randomness. However, we recommend the use of the best prediction approach (conditional expectation) that uses the additional information given by the phenotypic data in an optimal way, minimizes the mean square error of prediction, includes the contribution of LD, and maintains the structure of the genomic equivalent of the classical additive genetic variance.

## Appendix

### Genomic Best Linear Unbiased Prediction

In the REM $\left(\beta_{j}∼N\left(0,\sigma_{\beta }^{2}\right)\right)$ for the model

$$y=\mu 1_{n}+PX\beta +\varepsilon$$ (6)

we have that

$$y∼N\left(\mu 1_{n},\underset{:={\tilde{\Sigma }}^{-1}}{\underset{︸}{PXX^{⊤}P\sigma_{\beta }^{2}+\sigma_{\varepsilon }^{2}I_{n}}}\right).$$ (26)

The marker effect vector *β* cannot be estimated because it is a random variable. Henderson (1984) introduced the concept of the prediction of *β*, which refers to the estimation of the realized values of the random effects. The best linear unbiased predictor (BLUP) $\mu_{\beta |y}$ for *β* is given by $\mu_{\beta |y}=E\left[\beta |y\right]$ (Henderson 1984; Searle *et al.* 1992). The conditional expectation is the unique best predictor, *i.e.* it is unbiased and has minimal mean square error of prediction

$$E\left[{\left(\beta -g\left(y\right)\right)}^{⊤}\left(\beta -g\left(y\right)\right)\right]$$

within the whole set of functions *g* that depend on the data *y* (van der Vaart 2007).

The joint distribution of *y* and β equals

$$\left(\begin{array}{c} y\\ \beta \end{array}\right)∼N\left[\left(\begin{array}{c} \mu 1_{n}\\ 0 \end{array}\right),\left(\begin{array}{cc} {\tilde{\Sigma }}^{-1} & \sigma_{\beta }^{2}PX\\ \sigma_{\beta }^{2}X^{⊤}P & \sigma_{\beta }^{2}I_{p} \end{array}\right)\right]$$

and we obtain

$$\beta |y∼N\left(\sigma_{\beta }^{2}X^{⊤}P\tilde{\Sigma }\left(y-\mu 1_{n}\right),\sigma_{\beta }^{2}I_{p}-\sigma_{\beta }^{2}X^{⊤}P\tilde{\Sigma }PX\sigma_{\beta }^{2}\right),$$ (27)

see *e.g.* Kotz *et al.* (2000). Consequently, the BLUP equals

$$\mu_{\beta |y}=\sigma_{\beta }^{2}X^{⊤}P\tilde{\Sigma }\left(y-\mu 1_{n}\right)$$ (28)

and is linear in *y*. The conditional variance-covariance matrix of *β* equals

$${\text{Σ}}_{\beta |y}:=\text{Cov}\left(\beta |y\right)\overset{\left(27\right)}{=}\sigma_{\beta }^{2}I_{p}-\sigma_{\beta }^{2}X^{⊤}P\tilde{\Sigma }PX\sigma_{\beta }^{2},$$ (29)

and the variance-covariance matrix of the BLUP $\mu_{\beta |y}$ equals

$$\begin{array}{l} {\text{Σ}}_{\mu_{\beta |y}}:=\text{Cov}\left(\mu_{\beta |y}\right)\\ =\text{Cov}\left(E\left[\beta |y\right]\right)\\ =\text{Cov}\left(\beta \right)-E\left[\text{Cov}\left(\beta |y\right)\right]\\ \overset{\left(29\right)}{=}\sigma_{\beta }^{2}X^{⊤}P\tilde{\Sigma }PX\sigma_{\beta }^{2}. \end{array}$$ (30)

The actual estimation of the parameters in model (6) with the BLUP-method is a two-stage procedure (Das *et al.* 2004). In the first stage, a BLUE for the fixed quantities and a BLUP for the random variables are derived. However, they involve the variance components $\sigma_{\beta }^{2}$ and $\sigma_{\varepsilon }^{2}$ as unknown parameters. In a second stage, these parameters are replaced by estimates, and the estimators for the BLUE and the BLUP are referred to as empirical BLUE (eBLUE) and empirical BLUP (eBLUP) (see Kackar and Harville 1984; Jiang 1999). Investigations on the properties of the eBLUE and the eBLUP are very complex (Searle *et al.* 1992), and often only approximate results are obtained (Kackar and Harville 1984; Jiang 1999; Das *et al.* 2004).

**Table A.1:** Overview of prediction approaches for the random additive genomic variance in the random-effects model with the gBLUP-method $\left(s_{g,B}^{2}=\frac{1}{n-1}\beta^{⊤}X^{⊤}PBPX\beta \right)$

	Unconditional expectation	Best prediction
General Formula	$E\left[s_{g,B}^{2}\right]=\frac{1}{n-1}\sigma_{\beta }^{2}\text{tr}\left(X^{⊤}PBPX\right)$	$\text{BP}\left(s_{g,B}^{2}\right)=\frac{1}{n-1}\text{tr}\left(X^{⊤}PBPX\left[\mu_{\beta |y}\mu_{\beta |y}^{⊤}+{\text{Σ}}_{\beta |y}\right]\right)$
Current population	$V=\sigma_{\beta }^{2}\text{tr}\left({\hat{\Sigma }}_{X}\right)$	$W=\text{tr}\left({\hat{\Sigma }}_{X}\left[\mu_{\beta |y}\mu_{\beta |y}^{⊤}+{\text{Σ}}_{\beta |y}\right]\right)$
Base population	$V^{\text{*}}=\sigma_{\beta }^{2}\text{tr}\left({\hat{\Sigma }}_{X^{\text{*}}}\right)$	$W^{\text{*}}=\text{tr}\left({\hat{\Sigma }}_{X^{\text{*}}}\left[\mu_{\beta |y}\mu_{\beta |y}^{⊤}+{\text{Σ}}_{\beta |y}\right]\right)$
Features	• Best approximation of the additive genomic variance in the absence of information	• Best approximation of the additive genomic variance using additional information given by phenotypic values (optimal adaptation to the data by application of the conditional or posterior expectation)
	• No inclusion of LD	• Explicit inclusion of LD
		• Orthogonal decomposition of the phenotypic variance in the current population (unique definition of the heritability)
		• Genomic equivalent of the additive genetic variance

**X**, matrix of marker genotypes; **P**, matrix for column-wise mean-centering; **B**, positive semidefinite matrix; $\sigma_{\beta }^{2},$ variance component of the marker effects *β*; $\mu_{\beta |y},$ BLUP of *β*; ${\text{Σ}}_{\beta |y},$ conditional covariance matrix of *β* given the phenotypic data *y*; ${\hat{\Sigma }}_{X},$ sample variance-covariance matrix of the marker genotypes in the current population; ${\hat{\Sigma }}_{X^{\text{*}}},$ sample variance-covariance matrix of the marker genotypes in the base population.

**Table A.2:** Overview of prediction approaches for the random additive genomic variance in the random-effects model with the gBLUP-method in the equivalent version of the linear model $\left(s_{g,B}^{2}=\frac{1}{n-1}g^{⊤}Bg\right)$

	Unconditional expectation	Best prediction
General formula	$E\left[s_{g,B}^{2}\right]=\frac{1}{n-1}\sigma_{g}^{2}\text{tr}\left(BG\right)$	$\text{BP}\left(s_{g,B}^{2}\right)=\frac{1}{n-1}\text{tr}\left(B\left[\mu_{g|y}\mu_{g|y}^{⊤}+{\text{Σ}}_{g|y}\right]\right)$
Current population	$V=\frac{1}{n-1}\sigma_{g}^{2}\text{tr}\left(G\right)$	$W=\frac{1}{n-1}\text{tr}\left(\left[\mu_{g|y}\mu_{g|y}^{⊤}+{\text{Σ}}_{g|y}\right]\right)$
Base population	$V^{\text{*}}=\frac{1}{n-1}\sigma_{g}^{2}\text{tr}\left(PR^{-0.5}GR^{-0.5}\right)$	$W^{\text{*}}=\frac{1}{n-1}\text{tr}\left(PR^{-0.5}\left[\mu_{g|y}\mu_{g|y}^{⊤}+{\text{Σ}}_{g|y}\right]R^{-0.5}\right)$
Features	• Best approximation of the additive genomic variance in the absence of information	• Best approximation of the additive genomic variance using additional information given by phenotypic values (optimal adaptation to the data by application of the conditional or posterior expectation)
	• No inclusion of LD	• Explicit inclusion of marker LD
	• Transformation with GRM: $\sigma_{g}^{2}$ replaces $V^{\text{*}}$	• Orthogonal decomposition of the phenotypic variance in the current population (unique definition of the heritability)
		• Genomic equivalent of the additive genetic variance

**G**, genomic relationship matrix; **P**, matrix for column-wise mean-centering; **B**, positive semidefinite matrix; $\sigma_{g}^{2},$ variance component of the genomic values *g*; $\mu_{g|y},$ the BLUP of *g*; ${\text{Σ}}_{g|y},$ conditional covariance matrix of *g* given the phenotypic data *y*; **R**, relationship matrix.

Assume that we are provided with estimators for the variance components using, *e.g.*, REML (Patterson and Thompson 1971; Corbeil and Searle 1976; Searle *et al.* 1992). These estimated variance components are nonlinear functions of the data *y*, and, consequently, the eBLUE

$$\hat{\mu }=\frac{1_{n}^{⊤}{\left(PXX^{⊤}P{\hat{\sigma }}_{\beta }^{2}+{\hat{\sigma }}_{\varepsilon }^{2}I_{n}\right)}^{-1}y}{1_{n}^{⊤}{\left(PXX^{⊤}P{\hat{\sigma }}_{\beta }^{2}+{\hat{\sigma }}_{\varepsilon }^{2}I_{n}\right)}^{-1}1_{n}}$$

for the intercept and the eBLUP

$${\hat{\mu }}_{\beta |y}={\hat{\sigma }}_{\beta }^{2}X^{⊤}P{\left(PXX^{⊤}P{\hat{\sigma }}_{\beta }^{2}+{\hat{\sigma }}_{\varepsilon }^{2}I_{n}\right)}^{-1}\left(y-\hat{\mu }\right),$$ (31)

for the marker effects *β* are not even linear in the data *y* anymore (despite their naming). The unbiasedness of the eBLUE and the eBLUP can be asserted if the estimated variance components ${\hat{\sigma }}_{\beta }^{2}$ and ${\hat{\sigma }}_{\varepsilon }^{2}$ are non-negative, even functions in *y*, translation-invariant, and if the expectations of the eBLUE and eBLUP are finite (Kackar and Harville 1984). When using REML estimates for the variance components, these requirements are satisfied and the eBLUE $\hat{\mu }$ and the eBLUP ${\hat{\mu }}_{\beta |y}$ are bias-free estimators for *μ* and *β* (Jiang 1999).

Conditional on the estimation of the variance components (ignoring the randomness in the second stage of the estimation of the eBLUP), the variance-covariance matrix of the eBLUP ${\hat{\mu }}_{\beta |y}$ equals

$$\begin{array}{l} {\text{Σ}}_{{\hat{\mu }}_{\beta |y}}:=\text{Cov}\left({\hat{\mu }}_{\beta |y}|\sigma_{\beta }^{2}={\hat{\sigma }}_{\beta }^{2},\sigma_{\varepsilon }^{2}={\hat{\sigma }}_{\varepsilon }^{2}\right)\\ =\sigma_{\beta }^{2}X^{⊤}P\tilde{\Sigma }\text{Cov}\left(y-\hat{\mu }1_{n}|\sigma_{\beta }^{2}={\hat{\sigma }}_{\beta }^{2},\sigma_{\varepsilon }^{2}={\hat{\sigma }}_{\varepsilon }^{2}\right)\tilde{\Sigma }PX\sigma_{\beta }^{2}=\sigma_{\beta }^{2}X^{⊤}P\tilde{\Sigma }PX\sigma_{\beta }^{2}-\frac{\sigma_{\beta }^{2}X^{⊤}P\tilde{\Sigma }1_{n}1_{n}^{⊤}\tilde{\Sigma }PX\sigma_{\beta }^{2}}{1_{n}^{⊤}\tilde{\Sigma }1_{n}}, \end{array}$$ (32)

because

$$\text{Cov}\left(y-\hat{\mu }1_{n}|\sigma_{\beta }^{2}={\hat{\sigma }}_{\beta }^{2},\sigma_{\varepsilon }^{2}={\hat{\sigma }}_{\varepsilon }^{2}\right)=$$

$$=\text{Cov}\left(y\right)-2\text{Cov}\left(y,\hat{\mu }|\sigma_{\beta }^{2}={\hat{\sigma }}_{\beta }^{2},\sigma_{\varepsilon }^{2}={\hat{\sigma }}_{\varepsilon }^{2}\right)1_{n}^{⊤}$$

$$+1_{n}\text{Cov}\left(\hat{\mu }|\sigma_{\beta }^{2}={\hat{\sigma }}_{\beta }^{2},\sigma_{\varepsilon }^{2}={\hat{\sigma }}_{\varepsilon }^{2}\right)1_{n}^{⊤}$$

$$={\tilde{\Sigma }}^{-1}-2\text{Cov}\left(y\right)\frac{\tilde{\Sigma }1_{n}}{1_{n}^{⊤}\tilde{\Sigma }1_{n}}1_{n}^{⊤}+1_{n}\frac{1_{n}^{⊤}\tilde{\Sigma }{\tilde{\Sigma }}^{-1}\tilde{\Sigma }1_{n}}{{\left(1_{n}^{⊤}\tilde{\Sigma }1_{n}\right)}^{2}}1_{n}^{⊤}$$

$$={\tilde{\Sigma }}^{-1}-\frac{1_{n}1_{n}^{⊤}}{1_{n}^{⊤}\tilde{\Sigma }1_{n}}$$

holds.

We can transfer the results derived in model (6) to model

$$y=\mu 1_{n}+g+\varepsilon$$ (1)

by using their equivalence in distribution. The gBLUP for *g* equals

$$\begin{array}{l} \mu_{g|y}:=E\left[g|y\right]=E\left[PX\beta |y\right]\\ =PX\mu_{\beta |y}\\ \overset{\left(28\right)}{=}PX\sigma_{\beta }^{2}X^{⊤}P\tilde{\Sigma }\left(y-\mu 1_{n}\right)\\ \overset{\left(3\right)}{=}\sigma_{g}^{2}G{\left(G\sigma_{g}^{2}+\sigma_{\varepsilon }^{2}I_{n}\right)}^{-1}\left(y-\mu 1_{n}\right). \end{array}$$ (33)

The conditional variance-covariance matrix of *g* is obtained as

$$\begin{array}{l} {\text{Σ}}_{g|y}\text{ :=Cov}\left(g|y\right)\\ =PX\text{Cov}\left(\beta |y\right)X^{⊤}P\\ \overset{\left(29\right)}{=}PX\left(\sigma_{\beta }^{2}I_{p}-\sigma_{\beta }^{2}X^{⊤}P\tilde{\Sigma }PX\sigma_{\beta }^{2}\right)X^{⊤}P\\ \overset{\left(3\right)}{=}\sigma_{g}^{2}G-\sigma_{g}^{2}G{\left(G\sigma_{g}^{2}+\sigma_{\varepsilon }^{2}I_{n}\right)}^{-1}G\sigma_{g}^{2}, \end{array}$$ (34)

as well as the variance-covariance matrix of the BLUP $\mu_{g|y}$

$$\begin{array}{l} {\text{Σ}}_{\mu_{g|y}}:=\text{Cov}\left(\mu_{g|y}\right)\\ =PX\text{Cov}\left(\mu_{\beta |y}\right)X^{⊤}P\\ \overset{\left(\text{30}\right)}{=}PX\sigma_{\beta }^{2}X^{⊤}P\tilde{\Sigma }PX\sigma_{\beta }^{2}X^{⊤}P\\ \overset{\left(3\right)}{=}\sigma_{g}^{2}G{\left(G\sigma_{g}^{2}+\sigma_{\varepsilon }^{2}I_{n}\right)}^{-1}G\sigma_{g}^{2}. \end{array}$$ (35)

The variance-covariance matrix of the eBLUP equals

$$\begin{array}{l} {\text{Σ}}_{{\hat{\mu }}_{g|y}}:=\text{Cov}\left({\hat{\mu }}_{g|y}|\sigma_{g}^{2}={\hat{\sigma }}_{g}^{2},\sigma_{\varepsilon }^{2}={\hat{\sigma }}_{\varepsilon }^{2}\right)\\ =\text{Cov}\left(PX{\hat{\mu }}_{\beta |y}|\sigma_{\beta }^{2}={\hat{\sigma }}_{\beta }^{2},\sigma_{\varepsilon }^{2}={\hat{\sigma }}_{\varepsilon }^{2}\right)\\ =PX{\text{Σ}}_{{\hat{\mu }}_{\beta |y}}X^{⊤}P\\ \overset{\left(\text{32}\right)}{=}PX\left[\sigma_{\beta }^{2}X^{⊤}P\tilde{\Sigma }PX\sigma_{\beta }^{2}-\frac{\sigma_{\beta }^{2}X^{⊤}P\tilde{\Sigma }1_{n}1_{n}^{⊤}\tilde{\Sigma }PX\sigma_{\beta }^{2}}{1_{n}^{⊤}\tilde{\Sigma }1_{n}}\right]X^{⊤}P\\ \overset{\left(3\right)}{=}\sigma_{g}^{2}G\tilde{\Sigma }G\sigma_{g}^{2}-\frac{\sigma_{g}^{2}G\tilde{\Sigma }1_{n}1_{n}^{⊤}\tilde{\Sigma }G\sigma_{g}^{2}}{1_{n}^{⊤}\tilde{\Sigma }1_{n}}. \end{array}$$ (36)

### Theoretical Variances of the Genomic Values in the REM

We review three different definitions of the theoretical variance of the genomic values in the REM (marker genotypes random, marker effects random, or both random). We focus the following analysis on the linear model (6) because of the explicit separation of marker genotypes and marker effects. For simplicity, we focus on the genomic variance in the current population. The results for the base population are obtained by replacing the data-generating process *X* with $X^{\text{*}}$.

#### Random genotypes and random effects

If the marker genotypes as well as the marker effects are the source of genomic variation, we calculate the variance of the genomic value according to the law of total variance as:

Var(Xβ)=E[Var(Xβ|β)]+Var(E[Xβ|β])

$$=E\left[\beta^{⊤}{\text{Σ}}_{X}\beta \right]+\text{Var}\left(E\left[X\right]\beta \right)$$

$$=\text{tr}\left({\text{Σ}}_{X}E\left[\beta \beta^{⊤}\right]\right)+E\left[X\right]{\text{Σ}}_{\beta }E{\left[X\right]}^{⊤}$$

$$=E{\left[\beta \right]}^{⊤}{\text{Σ}}_{X}E\left[\beta \right]+\text{tr}\left({\text{Σ}}_{\beta }{\text{Σ}}_{X}\right)+E\left[X\right]{\text{Σ}}_{\beta }E{\left[X\right]}^{⊤}.$$

The unconditional expectation and the variance operator in the second line apply to the random marker effect vector *β*. Because of the model assumptions on the marker effects in Equation 5, and the mean-centered marker genotypes (E[X]=0), we obtain

$$\text{Var}\left(X\beta \right)=\sigma_{\beta }^{2}\text{tr}\left({\text{Σ}}_{X}\right)$$ (37)

with the interpretation as the variance of a randomly sampled (representative) individual for a trait with random effects.

#### Fixed genotypes and random effects

If the genomic variation is caused by the marker effects only, and the marker genotypes are fixed, then the *n*-vector of genomic values is normally distributed:

$$g=PX\beta ∼N\left(0,PXX^{⊤}P\sigma_{\beta }^{2}\right).$$ (38)

In order to obtain an average theoretical variance of the individuals in the sample, we calculate the mean trace of the variance-covariance matrix of the genomic values:

$$\begin{array}{l} \frac{1}{n}\text{tr}\left(\text{Cov}\left(PX\beta \right)\right)=\frac{1}{n}\sigma_{\beta }^{2}\text{tr}\left(PXX^{⊤}P\right)\\ =\frac{n-1}{n}\sigma_{\beta }^{2}\text{tr}\left({\hat{\Sigma }}_{X}\right). \end{array}$$ (39)

This approximately equals the variance of a randomly sampled individual for a randomly sampled trait (see Equation 37). Even when the marker genotypes are fixed, their sample variance-covariance matrix contributes to the theoretical variance of the genomic values when averaging over the individuals in the sample.

#### Random genotypes and fixed effects

Probably the most common assumption on the nature of the genome is that the marker genotypes are random, whereas the marker effects are fixed (Falconer and Mackay 1996). In order to translate these assumption to the variance of the genomic values in the REM, we have to condition on the marker effects (*i.e.* we fix them). Then, the theoretical variance of the genomic values of a individual with random marker genotypes (representative individual), and with fixed marker effects, equals

$$\begin{array}{l} \text{Var}\left(X\beta |\beta \right)=\beta^{⊤}{\text{Σ}}_{X}\beta \\ =\sum_{j=1}^{p}\beta_{j}\text{Var}\left(X_{j}\right)+\sum_{i=1}^{p}\sum_{\begin{array}{l} j=1\\ j\neq i \end{array}}^{p}\beta_{i}\beta_{j}\text{Cov}\left(X_{i},X_{j}\right), \end{array}$$ (40)

and describes the genomic equivalent of the definition of the additive genetic variance (Bulmer 1971; Falconer and Mackay 1996).

### Estimation of the Additive Genomic Variance in the REM

In the sections *The expectation of the additive genomic variance* and *Best prediction of the additive genomic variance*, we have introduced ways to predict the random additive genomic variance

$$s_{g,B}^{2}:=\frac{1}{n-1}g^{⊤}Bg=\frac{1}{n-1}\beta^{⊤}X^{⊤}PBPX\beta$$ (13)

in the REM, namely by using the unconditional expectation

$$\begin{array}{l} E\left[s_{g,B}^{2}\right]=\frac{1}{n-1}\sigma_{\beta }^{2}\text{tr}\left(X^{⊤}PBPX\right)\\ =\frac{1}{n-1}\sigma_{g}^{2}\text{tr}\left(BG\right) \end{array}$$ (14)

and the best predictor

$$\begin{array}{l} \text{BP}\left(s_{g,B}^{2}\right)=\frac{1}{n-1}\text{tr}\left(X^{⊤}PBPX\left[\mu_{\beta |y}\mu_{\beta |y}^{⊤}+{\text{Σ}}_{\beta |y}\right]\right)\\ =\frac{1}{n-1}\text{tr}\left(B\left[\mu_{\beta |y}\mu_{\beta |y}^{⊤}+{\text{Σ}}_{\beta |y}\right]\right). \end{array}$$ (21)

In the following, we introduce estimators for these quantities and investigate their properties.

#### Estimation of the unconditional expectation

For any given positive semidefinite matrix **B**, the unconditional expectation

$$\frac{1}{n-1}\sigma_{\beta }^{2}\text{tr}\left(X^{⊤}PBPX\right)$$

in model (6) can be estimated by

$$\frac{1}{n-1}{\hat{\sigma }}_{\beta }^{2}\text{tr}\left(X^{⊤}PBPX\right)$$

after having obtained an estimate ${\hat{\sigma }}_{\beta }^{2}$ of the variance component $\sigma_{\beta }^{2}$. In the equivalent model (1), we can estimate

$$\frac{1}{n-1}\sigma_{g}^{2}\text{tr}\left(BG\right)$$

by using

$$\frac{1}{n-1}{\hat{\sigma }}_{g}^{2}\text{tr}\left(BG\right)$$

for any positive semidefinite matrix **B**.

The specification of **B** as in the section *The expectation of the additive genomic variance* leads to the explicit form of the estimators

$$\hat{V}={\hat{\sigma }}_{\beta }^{2}\text{tr}\left({\hat{\Sigma }}_{X}\right)=\frac{1}{n-1}{\hat{\sigma }}_{g}^{2}\text{tr}\left(G\right),$$

$${\hat{V}}^{\text{*}}={\hat{\sigma }}_{\beta }^{2}\text{tr}\left({\hat{\Sigma }}_{X^{\text{*}}}\right)=\frac{1}{n-1}{\hat{\sigma }}_{g}^{2}\text{tr}\left(PR^{-0.5}GR^{-0.5}\right)$$

and

$${\hat{V}}_{\text{s}}^{\text{*}}=c{\hat{\sigma }}_{\beta }^{2}={\hat{\sigma }}_{g}^{2}.$$ (41)

#### Empirical best prediction

Because of equalities (Equations 29 and 30) and the variance-covariance matrix $\sum_{\beta }=\sigma_{\beta }^{2}I_{p}$ of *β*, we have that

$${\text{Σ}}_{\beta |y}={\text{Σ}}_{\beta }-{\text{Σ}}_{\mu_{\beta |y}}=\sigma_{\beta }^{2}I_{p}-{\text{Σ}}_{\mu_{\beta |y}}.$$

Consequently, the best predictor of $s_{g,B}^{2}$ defined in Equation 21 equals

$$\begin{array}{l} \text{BP}\left(s_{g,B}^{2}\right)=\frac{1}{n-1}\text{tr}\left(X^{⊤}PBPX\left[\mu_{\beta |y}\mu_{\beta |y}^{⊤}+{\text{Σ}}_{\beta |y}\right]\right)\\ =\frac{1}{n-1}\text{tr}\left(X^{⊤}PBPX\left[\mu_{\beta |y}\mu_{\beta |y}^{⊤}+{\text{Σ}}_{\beta }-{\text{Σ}}_{\mu_{\beta |y}}\right]\right)\\ =\frac{1}{n-1}\sigma_{\beta }^{2}\text{tr}\left(X^{⊤}PBPX\right)\\ +\frac{1}{n-1}\text{tr}\left(X^{⊤}PBPX\left[\mu_{\beta |y}\mu_{\beta |y}^{⊤}-{\text{Σ}}_{\mu_{\beta |y}}\right]\right)\\ =E\left[s_{g,B}^{2}\right]+L\left(y\right), \end{array}$$ (42)

where

$$\begin{array}{l} L\left(y\right):=\frac{1}{n-1}\text{tr}\left(X^{⊤}PBPX\left[\mu_{\beta |y}\mu_{\beta |y}^{⊤}-{\text{Σ}}_{\mu_{\beta |y}}\right]\right)\\ =\frac{1}{n-1}\text{tr}\left(B\left[\mu_{g|y}\mu_{g|y}^{⊤}-{\text{Σ}}_{\mu_{g|y}}\right]\right). \end{array}$$ (43)

We have partitioned the best predictor of $s_{g,B}^{2}$ into the unconditional expectation of $s_{g,B}^{2}$ and the random variable *L*, which is realized in the phenotypic data *y*. The random variable *L* specifies the adaption of the best predictor to the data and incorporates the contribution of (marker) LD. The expectation of *L* over all possible data *y* is 0 because

$$E\left[\mu_{\beta |y}\mu_{\beta |y}^{⊤}-{\text{Σ}}_{\mu_{\beta |y}}\right]=\text{Cov}\left(\mu_{\beta |y}\right)+E\left[\mu_{\beta |y}\right]E{\left[\mu_{\beta |y}\right]}^{⊤}-{\text{Σ}}_{\mu_{\beta |y}}=0.$$

The sign of the realization of *L* determines whether the best predictor is larger (positive weighted LD) or smaller (negative weighted LD) than the unconditional expectation.

The task of finding an eBP for $s_{g,B}^{2}$ is reduced to estimating the realized values of *L* because of the connection derived in Equation 42. We replace the BLUP and their variance-covariance matrix in Equation 43 by the eBLUP and their estimated variance-covariance matrix:

$$\hat{L}\left(y\right):=\frac{1}{n-1}\text{tr}\left(X^{⊤}PBPX\left[{\hat{\mu }}_{\beta |y}{\hat{\mu }}_{\beta |y}^{⊤}-{\hat{\Sigma }}_{{\hat{\mu }}_{\beta |y}}\right]\right).$$

We assume that we are provided with REML-estimators ${\hat{\sigma }}_{\beta }^{2}$ and ${\hat{\sigma }}_{\varepsilon }^{2}$ for the variance components. Then, we find

$$E\left[{\hat{\mu }}_{\beta |y}{\hat{\mu }}_{\beta |y}^{⊤}\right]=\text{Cov}\left({\hat{\mu }}_{\beta |y}\right)+E\left[{\hat{\mu }}_{\beta |y}\right]E{\left[{\hat{\mu }}_{\beta |y}\right]}^{⊤}$$

$$\overset{\left(\text{32}\right)}{=}{\text{Σ}}_{{\hat{\mu }}_{\beta |y}},$$

because the eBLUP is unbiased for *β*, *i.e.* $E\left[{\hat{\mu }}_{\beta |y}\right]=E\left[\beta \right]=0$ (Jiang 1999). Unfortunately, the unbiasedness of the estimated variance-covariance matrix of the eBLUP can be asserted only in a trivial way by conditioning on the estimated variance components:

$$E\left[{\hat{\Sigma }}_{{\hat{\mu }}_{\beta |y}}|\sigma_{\beta }^{2}={\hat{\sigma }}_{\beta }^{2},\sigma_{\varepsilon }^{2}={\hat{\sigma }}_{\varepsilon }^{2}\right]\overset{\left(32\right)}{=}{\text{Σ}}_{{\hat{\mu }}_{\beta |y}}.$$

Therefore, the expectation of $\hat{L}\left(y\right)$ (conditionally on the variance components) equals 0.

The same holds true for

$$\hat{L}\left(y\right)=\frac{1}{n-1}\text{tr}\left(B\left[{\hat{\mu }}_{g|y}{\hat{\mu }}_{g|y}^{⊤}-{\hat{\Sigma }}_{{\hat{\mu }}_{g|y}}\right]\right)$$

in the equivalent model, because the quantities ${\hat{\mu }}_{g|y}$ and ${\hat{\Sigma }}_{{\hat{\mu }}_{g|y}}$ are linear combinations of ${\hat{\mu }}_{\beta |y}$ and ${\hat{\Sigma }}_{{\hat{\mu }}_{g|y}}$ (see Equations 33 and 36).

Altogether, we can define the unbiased (conditionally on the estimated variance components) eBP

$$\text{eBP}\left(s_{g,B}^{2}\right):=\hat{E}\left[s_{g,B}^{2}\right]+\hat{L}\left(y\right)$$

$$=\frac{1}{n-1}{\hat{\sigma }}_{\beta }^{2}\text{tr}\left(X^{⊤}PBPX\right)$$

$$+\frac{1}{n-1}\text{tr}\left(X^{⊤}PBPX\left[{\hat{\mu }}_{\beta |y}{\hat{\mu }}_{\beta |y}^{⊤}-{\hat{\Sigma }}_{{\hat{\mu }}_{\beta |y}}\right]\right)$$

$$=\frac{1}{n-1}{\hat{\sigma }}_{g}^{2}\text{tr}\left(BG\right)+\frac{1}{n-1}\text{tr}\left(B\left[{\hat{\mu }}_{g|y}{\hat{\mu }}_{g|y}^{⊤}-{\hat{\Sigma }}_{{\hat{\mu }}_{g|y}}\right]\right)$$

for the additive genomic variance $s_{g,B}^{2}$.

The specification of B as in the section *Best prediction of the additive genomic variance* leads to the explicit form

$$\hat{W}:=\text{eBP}\left(s_{g}^{2}\right)=\hat{V}+\text{tr}\left({\hat{\Sigma }}_{X}\left[{\hat{\mu }}_{\beta |y}{\hat{\mu }}_{\beta |y}^{⊤}-{\hat{\Sigma }}_{{\hat{\mu }}_{\beta |y}}\right]\right)=\hat{V}+\frac{1}{n-1}\text{tr}\left(\left[{\hat{\mu }}_{g|y}{\hat{\mu }}_{g|y}^{⊤}-{\hat{\Sigma }}_{{\hat{\mu }}_{g|y}}\right]\right)$$

of the eBP for the additive genomic variance in the current population, and to the eBP

$${\hat{W}}^{\text{*}}:=\text{eBP}\left(s_{g*}^{2}\right)$$

$$={\hat{V}}^{\text{*}}+\text{tr}\left({\hat{\Sigma }}_{X^{\text{*}}}\left[{\hat{\mu }}_{\beta |y}{\hat{\mu }}_{\beta |y}^{⊤}-{\hat{\Sigma }}_{{\hat{\mu }}_{\beta |y}}\right]\right)$$

$$={\hat{V}}^{\text{*}}+\frac{1}{n-1}\text{tr}\left(PR^{-0.5}\left[{\hat{\mu }}_{g|y}{\hat{\mu }}_{g|y}^{⊤}-{\hat{\Sigma }}_{{\hat{\mu }}_{g|y}}\right]R^{-0.5}\right)$$

for the additive genomic variance in the base population.

Using the GRM **G** for a transformation to the base population is not well-defined because **G** is singular. However, because ${\hat{V}}_{\text{s}}^{\text{*}}$ (see Equation 41), is commonly used, we want to find an analogous formula for the eBP in this set-up.

Instead of calculating

$$G^{-0.5}{\hat{\mu }}_{g|y}=G^{-0.5}{\hat{\sigma }}_{g}^{2}G{\left(G{\hat{\sigma }}_{g}^{2}+{\hat{\sigma }}_{\varepsilon }^{2}I_{n}\right)}^{-1}\left(y-\hat{\mu }1_{n}\right)$$

and

$$G^{-0.5}{\hat{\Sigma }}_{{\hat{\mu }}_{g|y}}=G^{-0.5}\left[{\hat{\sigma }}_{g}^{2}G\hat{\tilde{\text{Σ}}}G{\hat{\sigma }}_{g}^{2}-\frac{{\hat{\sigma }}_{g}^{2}G\hat{\tilde{\text{Σ}}}1_{n}1_{n}^{⊤}\hat{\tilde{\text{Σ}}}G{\hat{\sigma }}_{g}^{2}}{1_{n}^{⊤}\hat{\tilde{\text{Σ}}}1_{n}}\right]G^{-0.5},$$

we use

$${\hat{\mu }}_{g|y}^{\text{*}}:={\hat{\sigma }}_{g}^{2}G^{0.5}{\left(G{\hat{\sigma }}_{g}^{2}+{\hat{\sigma }}_{\varepsilon }^{2}I_{n}\right)}^{-1}\left(y-\hat{\mu }1_{n}\right)$$

and

$${\hat{\text{Σ}}}_{{\hat{\mu }}_{g|y}}^{\text{*}}:={\hat{\sigma }}_{g}^{2}G^{0.5}\hat{\tilde{\text{Σ}}}G^{0.5}{\hat{\sigma }}_{g}^{2}-\frac{{\hat{\sigma }}_{g}^{2}G^{0.5}\hat{\tilde{\text{Σ}}}1_{n}1_{n}^{⊤}\hat{\tilde{\text{Σ}}}G^{0.5}{\hat{\sigma }}_{g}^{2}}{1_{n}^{⊤}\hat{\tilde{\text{Σ}}}1_{n}}$$

as substitutes. Then, we define

$${\hat{W}}_{\text{s}}^{\text{*}}={\hat{V}}_{\text{s}}^{\text{*}}+\frac{1}{n-1}\text{tr}\left(P\left[{\hat{\mu }}_{g|y}^{\text{*}}{\left({\hat{\mu }}_{g|y}^{\text{*}}\right)}^{⊤}-{\hat{\text{Σ}}}_{{\hat{\mu }}_{g|y}}^{\text{*}}\right]\right)$$

as an approximation of the eBP of the additive genomic variance in the base population when using the GRM for the transformation.
